# Supplementary material for: Effective cell membrane tension is independent of polyacrylamide substrate stiffness
Source: PNAS Nexus. 2022 Jan 6;2(1):pgac299. doi: 10.1093/pnasnexus/pgac299 (PMC9887938; doi:10.1093/pnasnexus/pgac299)
Supplement: pgac299_Supplemental_Files [file pgac299_supplemental_files.zip › PNASNEXUS-PNASNEXUS-2022-00563-T-s03.pdf]

Supplementary Material

Effective cell membrane tension is independent of polyacrylamide substrate stiffness

Eva Kreysing[1,*], Jeffrey Mc Hugh[2,3,*], Sarah K. Foster[1,4], Kurt Andresen[5], Ryan D. Greenhalgh[1], Eva K. Pillai[1], Andrea Dimitracopoulos[1], Ulrich F. Keyser[2,#], Kristian Franze[1,6,7,#]

1) Department of Physiology, Development and Neuroscience, University of Cambridge, Cambridge CB2 3DY, UK

2) Biological and Soft Systems, Maxwell Centre, Cavendish Laboratory, Cambridge CB3 0HE, UK

3) Neuroglial Interactions in Cerebral Physiopathology, CIRB, CNRS UMR 7241/INSERM U1050, Collège de France, Paris 75005, France

4) Systems Biology of Microbial Communities, Cluster of Excellence – CMFI, University of Tübingen, 72076 Tübingen, Germany

5) Department of Physics, Gettysburg College, Gettysburg, PA 17325, United States of America

6) Institute of Medical Physics, Friedrich-Alexander-Universität Erlangen-Nürnberg, 91052 Erlangen, Germany.

7) Max-Planck-Zentrum für Physik und Medizin, 91054 Erlangen, Germany

* These authors contributed equally.

# To whom correspondence should be addressed: *ufk20@cam.ac.uk* or kf284@cam.ac.uk

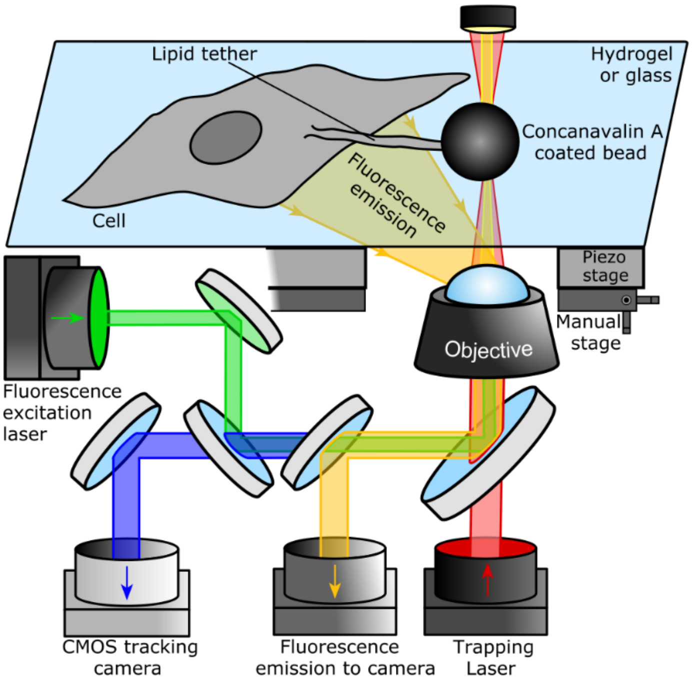

Figure S1: Schematic of the optical tweezers setup. The optical trap (OT) trapped a bead coated with concanavalin A. The bead was positioned close to a cell's membrane using a manual microscope stage. After approximately 5 seconds, the bead was moved away from the cell using a piezo stage. The deflection of the bead due to a membrane tether was recorded with a CMOS camera. When the pull was complete, fluorescence microscopy was used to visualise the tether (Figs. 2a, e).

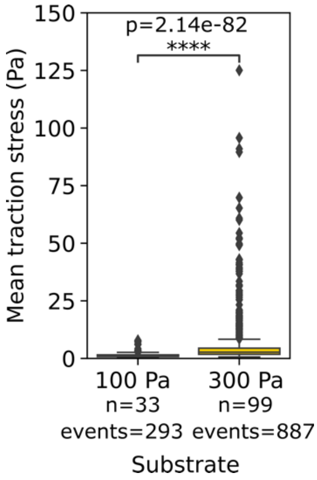

Figure S2: Traction forces of neuronal growth cones. The cells exerted higher traction forces on stiff hydrogels compared to softer hydrogels (two-tailed Mann-Whitney test). *n* describes the number of cells, whereas *events* counts the number force events that was analysed.

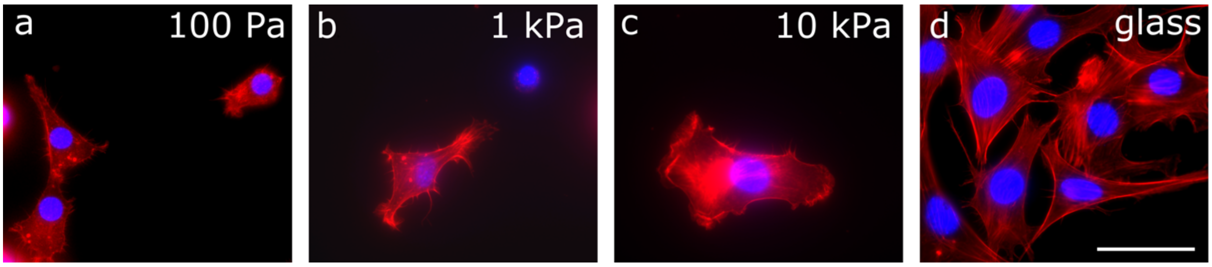

Figure S3: Immunostainings of fibroblasts on different substrates. Fibroblasts grown on hydrogels and glass, stained for actin (red) and nuclei (blue). Note the drastic change in morphology between hydrogel and glass substrates. Cells on glass exhibited pronounced stress fibres. Scale bar: 50µm.

Videos

Video S1 Tether Pull

The video shows a tether pull from an axon as described in the methods. The tether was pulled perpendicularly to the axon. The tether only slid slightly to the left during the pull. The scale bar is 10 µm, the video was recorded in real time at 4 fps. The video was sped up by 6.5x compared to real time.

Video S2 Sliding Tether

The video shows a tether pull from an axon as described in the methods. The tether was pulled almost perpendicularly to the axon. The tether slid towards the right during the pull. The scale bar is 10 µm, the video was recorded in real time at 8 fps. The video was sped up by 3.25x compared to real time.
